# Response of Gene Expression and Alternative Splicing to Distinct Growth Environments in Tomato

**DOI:** 10.3390/ijms18030475

**Published:** 2017-03-02

**Authors:** Guixiang Wang, Lin Weng, Meng Li, Han Xiao

**Affiliations:** 1 University of Chinese Academy of Sciences, 19A Yuquan Rd., Beijing 100049, China; gswang@sibs.ac.cn; 2 National Key Laboratory of Plant Molecular Genetics, CAS Center for Excellence in Molecular Plant Sciences, Institute of Plant Physiology and Ecology, Chinese Academy of Sciences (CAS), 300 Fenglin Rd., Shanghai 200032, China; lweng@sibs.ac.cn (L.W.); limeng22387@126.com (M.L.)

**Keywords:** alternative splicing, RNA sequencing, transcriptome, phenotypic plasticity, tomato

## Abstract

Phenotypic plasticity is the phenomenon that one particular genotype produces different phenotypes under different environmental conditions, but its underlying molecular and genetic mechanisms are poorly understood. Plastic traits may be under the control of genes whose expression is modulated by environmental cues. In this study, we investigated phenotypic plasticity in tomato (*Solanum lycopersicum*) and its ancestral species *S. pimpinellifolium* by comparing the global gene expression of young seedlings grown under two distinct growth conditions. Our results show that more than 7000 genes exhibited differential expression in response to environmental changes from phytotron to a plastic greenhouse, and 98 environmentally sensitive genes displayed the same patterns of expression response across the two tomato species. We also found that growth conditions had a remarkable impact on transcriptome complexity, attributable to alternative splicing (AS), in which 665 splice variants showed differential expression in response to the environmental changes. Moreover, more splice variants and AS events per gene were detected in plastic greenhouse-grown seedlings than their phytotron counterparts, and these seedlings also had higher percentages of intron retention events. The identification of the conserved environmentally-sensitive genes and the splice variants in this study will be useful for further analysis of gene regulation of environmental response in tomato and other crops.

## 1. Introduction

As sessile organisms, plants have to cope with changing environments during their life cycles. A key strategy for plants to adapt to environment perturbations is phenotypic plasticity, a phenomenon that a single genotype produces varied phenotypes in different environments [1]. Although phenotypic plasticity has a profound impact on ecology and agriculture, the underlying genetic and molecular mechanisms remain poorly understood. It is well known that the physiological and biochemical changes in plants under particular external stimuli are associated with altered gene expression. Phenotypic plasticity is likely achieved through differential gene expression in response to changing environments. Indeed, recent studies start to reveal the transcriptome plasticity of several plant species [2,3,4,5,6]. Among them, several global gene expression analyses have provided informative knowledge on transcriptome dynamics of crops grown under fluctuating field conditions [7,8,9]. A study in grapevine (*Vitis vinifera*) used vegetatively-propagated clones from one cultivar to compare transcriptome changes in response to changes in cultivation locations and climate conditions [7]. From this study, 5% of the grapevine protein coding genes were found to be environmentally sensitive with significantly enriched ontology categories of transcription regulation and protein translation as well as secondary metabolism. A comprehensive transcriptome analysis in rice suggests that field type and macroclimate impose dramatic effects on gene expression involved in photosynthesis and development, and among these environmental factors solar radiation and temperature have consistent effects on gene expression [9]. When tobacco leaves were subjected to different climate, soil and tillage conditions, about seven hundred genes were commonly modulated by the three factors as revealed by RNA sequencing (RNA-seq) [10].

Alternative splicing (AS) of precursor message RNA (pre-mRNA) is thought to be one of the major driving forces in the regulation of gene expression in plants [11]. AS diversifies transcriptome and expands protein coding capacity, having a profound impact on protein functionality, stability and expression levels [12,13,14,15]. In plants, 40%–63% of AS frequencies have been reported for the expressed multi-exon genes [16,17,18,19,20,21,22]. A survey on AS patterns by computational analysis of expressed sequence tags (EST), mRNA and short reads from public available RNA-seq data has also revealed that depending on species, 39.1%–70.4% of the genes with introns produced more than one splice variant in the nine plant species investigated [23]. Although for most AS events, their biological significance remains to be unraveled, current studies clearly suggest that AS-mediated regulation of gene expression plays an important role in plant growth and development.

Alternative splicing regulates many biological processes including plant response to changing environments [14,24,25,26,27,28]. Light is an energy resource for green plants, the adaptation of which to altered light conditions may cause massive changes in plant physiology [29]. When dark-grown seedlings are subjected to light treatments, not only about 20% of all the Arabidopsis genes were differentially expressed, but also changes were caused in several hundred AS events [30]. Intron retention is the most abundant AS type in plants [31], which frequently yields transcripts containing premature termination codons (PTC) that are rapidly degraded by the cellular nonsense-mediated mRNA decay (NMD) machinery. Certain environmental stresses induce AS variants from intron retention [30,32,33]. However, light seems to promote the production of protein-encoding splice variants at the expense of PTC transcripts [30]. Furthermore, AS is modulated by developmental cues and environmental conditions [21,34,35,36,37,38,39]. Most of the splicing factor SR (serine/arginine-rich) genes also undergo AS and produce two or more splice variants [40]. The AS pattern of the SR30 gene in Arabidopsis varies among developmental stages and its expression level is impacted by stress conditions [41]. Therefore, AS may play a role in shaping the transcriptome dynamic under fluctuating environmental conditions.

**Figure 1:**
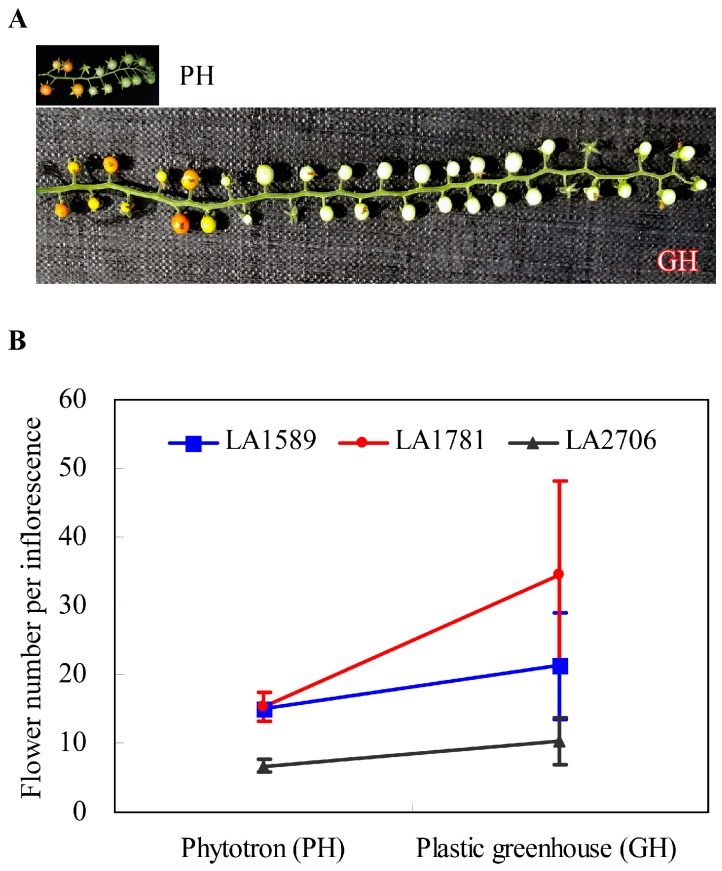
Plasticity of inflorescence development observed in three tomato accessions. (**A**) representative images showing plasticity of inflorescence development in *Solanum pimpinellifolium* LA1781. Inflorescences of LA1781 plants grown in a plastic greenhouse (GH) were much longer and harbored more flowers, compared to those of phytotron (PH) plants. The inflorescence (lower panel) was from three-months-old GH plants transplanted in mid-March 2016; (**B**) variations in plasticity of inflorescence development measured by flower number in LA1589, LA1781 and LA2706 (Moneymaker). Flower numbers were counted on 30–60 inflorescences of at least 10 (PH) or 30 (GH) plants. Data are represented by mean ± standard deviations (SD).

Tomato (*Solanum lycopersicum*) is an important vegetable crop growing world-wide. A considerable portion of tomatoes are grown in greenhouses world-wide [42,43]. These off-season greenhouse tomatoes usually are of relative lower quality than in-season field-grown ones, likely due to environmental differences, especially the dosage difference in ultraviolet (UV) radiation [42]. We noticed drastic variations in developmental plasticity among certain tomato accessions when grown in phytotron (temperature and humidity are well controlled) and a plastic greenhouse (solar radiation and without air-condition). For example, the cultivar Moneymaker showed a small change in flower number per inflorescence, whereas some *Solanum pimpenillifolium* accessions displayed strong inflorescence plasticity (Figure 1). Although the genetic and molecular basis underlying the differences in inflorescence plasticity among different tomato accessions remains to be unraveled, we hypothesize that transcriptional and/or posttranscriptional regulation on particular pathways might associate with this phenotypic plasticity because as mentioned above, environments impose significant impact on transcriptome dynamics. Before dissecting the genetic basis of inflorescence plasticity in these tomato accessions, we want to understand the transcriptome complexity of tomato seedlings grown in the two distinct conditions. In this study, we assessed, by RNA-seq, genome-wide gene expression of tomato seedlings and alternative splicing of pre-mRNA in response to distinct growth environments. Our results show that thousands of genes responded to environmental changes from phytotron to a plastic greenhouse in both the cultivated tomato and its closest wild relatives *S. pimpinellifolium*. There were also remarkable differences in AS pattern, including the number of AS events per gene and the frequencies of AS types between the two growth conditions. Moreover, the identification of the 98 common environmentally-sensitive genes across species provides a useful resource for functional genomics studies of environmentally regulated processes.

## 2. Results

### 2.1. Characterization of Alternative Splicing Events in Tomato Seedlings Growing in Two Distinct Environments

To understand the transcriptome dynamic of tomato seedlings in response to environmental conditions, RNA-seq analyses were conducted on two *S. pimpenillifolium* accessions—LA1589 and LA1781—and one cultivated tomato—LA2706 (Moneymaker)—which showed different phenotypic plasticity when grown in phytotron (denoted as PH, 16/8 h, light/dark; 20–25 °C) and a plastic greenhouse (denoted as GH, a standard commercial practice with solar radiation), respectively. RNA-seq libraries of three biological replicates per genotype were constructed using total RNA isolated from young (4–5 days post germination) seedlings and sequenced at 2 × 100 bp length by an Illumina Hiseq2500 machine. In total, 221.7 million read pairs were obtained from the 18 libraries with an average of 24.6 million sequence reads for each seedling sample (12.3 million read pairs) (Table S1). After quality check and removal of low quality reads and adapter sequences, clean reads were mapped to the reference genome ITAG2.5 by Tophat [44]. Then, we used the Cuffllinks program to assemble transcripts from the mapped reads, to estimate transcript abundance, and to identify differentially expressed (DE) genes and isoforms [44]. In total, 35,394 genes/loci were assembled and 2278 of these assembled genes were novel, which were not annotated in the current version (ITAG2.4) of the Heinz1706 reference genome. Among them, 23,974 genes (21,758 annotated in ITAG2.4) were considered to be expressed when a cutoff of FPKM (Fragments Per Kilobase of exon per Million fragments mapped) >0.5 (at least in one genotype and one growth condition) was applied. The numbers of expressed genes were similar among the three genotypes and between the two growth conditions; Moneymaker seedlings grown in phytotron had slightly fewer genes expressed (21,106), whereas LA1589 had several hundred more genes expressed in GH seedlings (21,952).

**Figure 2:**
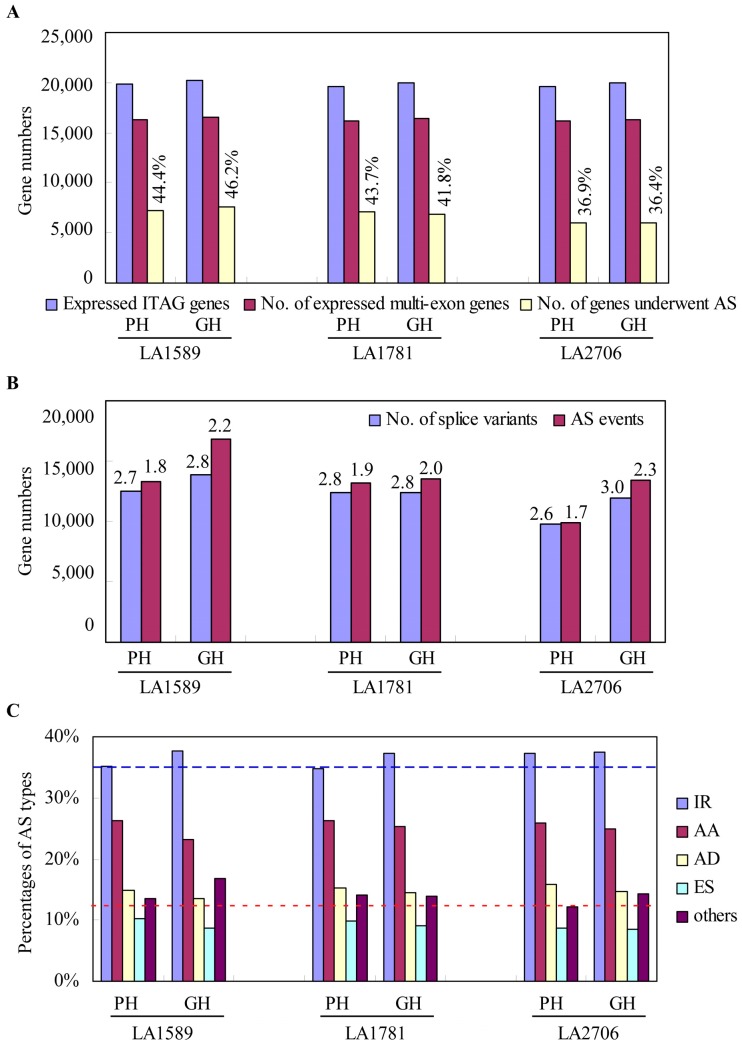
Alternative splicing (AS) response to distinct growth environments. (**A**) The number of expressed genes, genes with multi-exons and those that underwent AS in the seedlings grown under GH and PH conditions. AS frequencies were shown in the above the bar charts representing the number of genes that underwent AS; (**B**) The number of splice variants and AS events predicted. The numbers above the bar charts indicated the number of splice variants and AS events per gene; (**C**) Frequencies of AS types in transcriptome of GH and PH seedlings of LA1589, LA1781 and LA2706. The blue and red dotted lines indicate the lowest frequencies of intron retention (IR) and “others” types, respectively.

Alternative splicing (AS) of pre-mRNA is prevalent in eukaryotes and regulates gene expression during plant growth and development and in response to stresses [28]. We first investigated how AS responded to environmental changes in the three genotypes. Comparing to the predicted reference transcripts (ITAG2.4), we detected a total of 29,369 transcripts that possibly resulted from novel splicing junctions, which accounted for 42.1% of all assembled transcripts. These splice variants were from 10,410 multi-exon genes, meaning that there were 59.5% genes that underwent AS in these seedlings. The frequencies of the expressed multi-exon genes that underwent AS were slightly affected by genotypes—more genes produced splice variants in the two *S. pimpinellifolium* accessions—LA1589 and LA1781—than the cultivar Moneymaker. LA1589 and LA1781 had 41.8%–46.2% of the expressed multi-exon genes that underwent AS compared to the frequencies of 36.4% and 36.9% in the cultivated tomato Moneymaker under GH and PH conditions (Figure 2A). However, the numbers of isoforms per gene were similar among the three genotypes and between the two growth conditions, except the expressed multi-exon genes produced slightly more isoforms on average (3.0 isoforms per gene) in Moneymaker seedlings under GH condition (Figure 2B).

Then, using the online tool ASTALAVISTA, we extracted all AS events predicted from the GTF (gene transfer format) files generated by Cufflinks from the mapped reads of LA1589, LA1781 and Moneymaker seedlings. In total, 56,817 AS events were extracted and on average there were 5.46 events per gene. Interestingly, more AS events were detected in GH seedlings than their PH counterparts, suggesting that alternative splicing responds to environmental changes in tomato and certain environmental factors enhance AS. Further categorizing these AS events, we found intron retention (IR) is most abundant (34.7%–37.7%), followed by alternative splice donor (AD, 23.2%–26.3%), alternative splice acceptor (AA, 13.6%–15.9%), and exon skipping (ES) was the least abundant type (8.5%–10.2%) (Figure 2C). There was also a considerable portion of AS variants that resulted from complex events, in which more than one of the above-mentioned AS events were involved (classed as others, 12.2%–16.8%). For each genotype, GH seedlings had higher frequencies of the IR and “others” types, and accordingly there were relatively fewer AA, AD and ES types in PH seedlings.

### 2.2. Transcriptional Response to Environmental Changes

**Figure 3:**
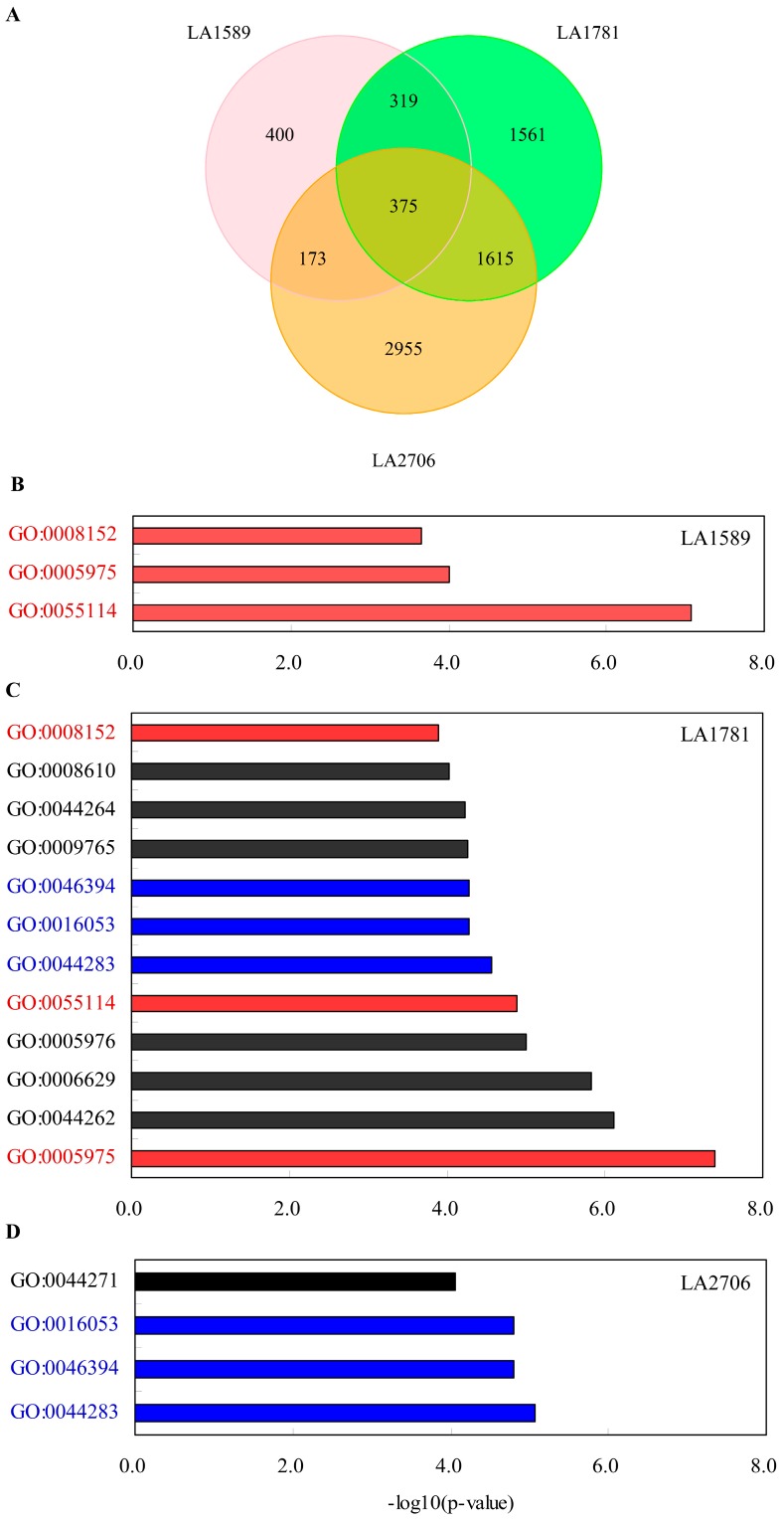
Gene ontology analysis of differentially expressed genes between GH and PH conditions. (**A**) Venn diagram of genes showing differential expression in response to different growth conditions; (**B**–**D**) Gene Ontology term analysis of genes responding to different growth conditions in LA1589 (**B**); LA1781 (**C**) and LA2706 (**D**), respectively. Enriched GO terms (adjusted *p*-value < 0.05) include carbohydrate metabolism (GO:0005975), cellular carbohydrate metabolism (GO:0044262), lipid metabolism (GO:0006629), polysaccharide metabolism (GO:0005976), oxidation reduction (GO:0055114), small molecule biosynthesis (GO:0044283), organic acid biosynthesis (GO:0016053), carboxylic acid biosynthesis (GO:0046394), photosynthesis, light harvesting (GO:0009765), cellular polysaccharide metabolism (GO:0044264), lipid biosynthesis (GO:0008610), metabolism (GO:0008152), and cellular nitrogen compound biosynthesis (GO:0044271). Shared GO terms between genotypes were highlighted in red or blue and those not shared were in black.

**Figure 4:**
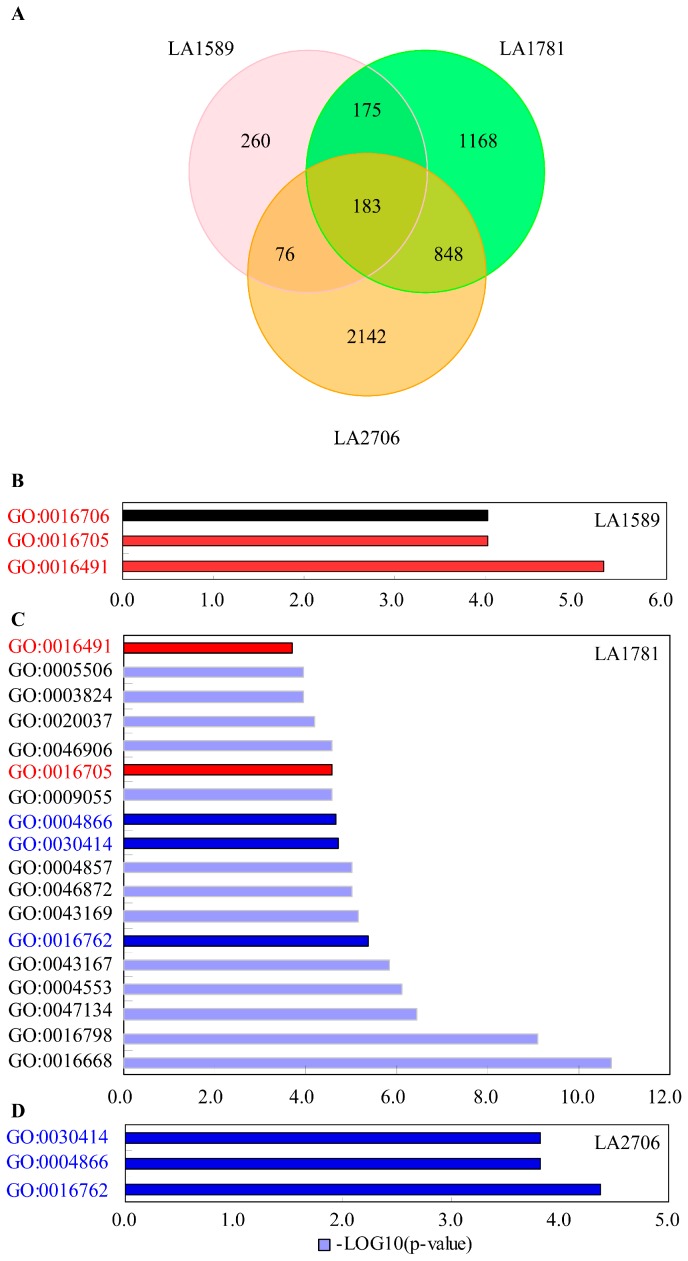
Gene ontology analysis of differentially expressed isoforms between GH and PH conditions. (**A**) Venn diagram of isoforms showing differential expression in response to different growth conditions; (**B**–**D**) Gene Ontology term analysis of genes with isoforms responding to different growth conditions in LA1589 (**B**); LA1781 (**C**) and LA2706 (**D**), respectively. Enriched GO terms (adjusted *p*-value < 0.05) include oxidoreductase activity (GO:0016705, GO:0016706, GO:0016491 and GO:0016668), iron ion binding (GO:0005506), catalytic activity (GO:0003824), heme binding (GO:0020037), tetrapyrrole binding (GO:0046906), electron carrier activity (GO:0009055), endopeptidase inhibitor activity (GO:0004866), peptidase inhibitor activity (GO:0030414), enzyme inhibitor activity (GO:0004857), metal ion binding (GO:0046872), cation binding (GO:0043169), xyloglucan:xyloglucosyl transferase activity (GO:0016762), ion binding (GO:0043167), hydrolase activity (GO:0004553 and GO:0016798), and protein-disulfide reductase activity (GO:0047134). Shared GO terms between genotypes were highlighted in red or dark blue, and those not shared were in light blue or black.

Because many splice variants were detected in these RNA samples, to better understand the transcriptome changes in response to changing environments, we investigated expression changes by growth conditions at gene and isoform levels. Both of the differentially expressed genes and isoforms were selected by Cufflinks with a cutoff of false discovery rate at 0.05 (adjusted *p*-value < 0.05). There was a total of 7398 DE genes between GH and PH seedlings from the three tomato genotypes (Tables S2–S4). Moneymaker had the most DE genes (5118), followed by LA1781 (3870) and LA1589 (1217). At the isoform level, 4852 DE isoforms from 4376 genes were identified (Table S5). Similarly, more isoforms were differentially expressed in Moneymaker seedlings (3249), followed by the two *S. pimpinellifolium* accessions LA1781 (2374) and LA1589 (694). Furthermore, we found that there were 375 genes and 183 isoforms showing differential expression in response to growth environmental changes in all of the three genotypes, and additional numbers of DE genes and isoforms were shared by two of the three genotypes (Figure 3A and Figure 4A). There was a noticeable difference in the number of genes showing differential expression at gene and isoform levels (7398 vs. 4376), implying that for some genes the abundance of isoform(s) may affect gene expression considerably.

We then conducted an enrichment analysis of gene ontology (GO) terms on the genes differentially expressed at gene and isoform levels in LA1589, LA1781 and Moneymaker. Singular enrichment analysis (SEA) of GO terms was performed by AgriGO [45], and over-represented GO terms were selected with FDR cutoff at 0.05 (adjusted *p*-value < 0.05). The analysis revealed that, at both gene and isoform levels, much more functional categories were over-represented in DE genes and isoforms of LA1781 than those of LA1589 and Moneymaker (Figure 3B–D and Figure 4B–D). Almost all the enriched GO terms in LA1589 and Moneymaker were also over-represented in LA781, except the functional category of the cellular nitrogen compound biosynthetic process (GO:0044271), which only was enriched in the DE genes identified in Moneymaker. We did not find any over-represented functional categories shared by the three tomato accessions. Overall, the enriched GO terms were similar between the DE genes and isoforms found in LA1781 and Moneymaker; most functional categories that were over-represented in the DE genes also were enriched in DE isoforms. However, the functional category of photosynthesis (GO:0009765) were over-represented in the DE isoforms, but not in the DE genes of LA1781 and Moneymaker, suggesting that alternative splicing of a set of genes involved in photosynthesis was responsive to the changing growth conditions.

### 2.3. Identification of Environment-Regulated Genes in Tomato

As shown in Figure 3, 375 genes showed differential expression in response to growth condition changes. When further applying a cutoff of an at least two-fold change at transcript levels, there were 98 out of the 375 DE genes whose expression was consistently higher or lower in all GH seedlings than their PH counterparts (Table S6). We considered the 98 DE genes as putative environmentally-sensitive tomato genes that may play important roles in adaptation to changing environments. Fourteen of these environmentally-sensitive genes are involved in photosynthesis or light signaling pathways (Table 1). Unexpectedly, all the nine environmentally-sensitive genes involved in photosynthetic light harvesting, electron transfer and carbon fixation were less abundant in GH seedlings, likely due to transcriptional repression by high light, a phenomenon also observed in bamboo (*Phyllostachys edulis*) [46]. Of the nine photosynthetic genes, three (*Solyc06g069730*, *Solyc05g056050* and *Solyc12g006140*) encode light-harvesting chlorophyll complex (LHC) subunits Lhca4, Lhca1 and Lhcb2.1, two (*Solyc08g006930* and *Solyc11g068430*) encode photosystem I subunit K (PsaK) and the electron transfer protein ferredoxin (FD3), respectively. The other four genes encode enzymes including two pyruvate decarboxylases, Fe superoxide dismutase (FSD2) and NADP-malic enzyme (NADP-ME4). Only three genes, likely involved in light-related signaling pathways, had more abundant transcripts in PH seedlings; they encode asparagine synthase (*Solyc06g007180*), an R2R3-type MYB (myeloblastosis) transcription factor (*Solyc05g008240*), and a protein shared high similarity to *Arabidopsis* cryptochrome-interacting basic-helix-loop-helix 1 (CIB1) (*Solyc01g109700*), respectively.

More AS events were detected in GH seedlings than those grown under PH condition (Figure 2B), suggesting that AS likely happened more frequently under GH condition. *Solyc09g008790* encodes a serine/threonine protein kinase highly similar to Arabidopsis SR protein-specific kinase 4 (SRPK4) that phosphorylates the SR protein RSp31 [8]. Higher *Solyc09g008790* expression detected in GH seedlings suggests that the splicesome machinery was regulated at transcription level. Then, we checked the expression pattern of splice variants to identify those whose expression is modulated by the environment. There were 665 splice variants having significantly altered expression in response to different growth conditions, of which 32, 270 and 467 splice variants had significantly different expression levels between GH and PH conditions in LA1589, LA1781 and Moneymaker, respectively (Table S7). However, compared to the number of genes showing differential expression in response to different growth conditions, there were only five splice variants with significantly different expression levels in all the three genotypes. The five variants are from five annotated genes: *Solyc03g083400* encoding a CCT (CONSTANS, CO-like, and TOC1) motif family protein, *Solyc05g008240* (an R2R3-type MYB transcription factor), *Solyc06g053710* (ethylene receptor ETR4), *Solyc02g078500* (Aluminum-induced protein-like) and *Solyc01g007350* (putative photosystem I reaction center subunit VIII). In addition, a splice variant of *Solyc06g059800* encoding Protein-P-II uridylyltransferase was also considered to be differentially expressed in the three genetic backgrounds because it had significantly higher expression (adjusted *p*-value < 0.05) in GH seedlings of LA1781 and Moneymaker, and was also more abundant (2.4-fold, adjusted *p*-value = 0.067) in the GH seedlings of LA1589. All the six genes showed differential gene expression, indicating that expression fluctuations of these splice variants were highly associated with gene expression levels.

**Figure 5:**
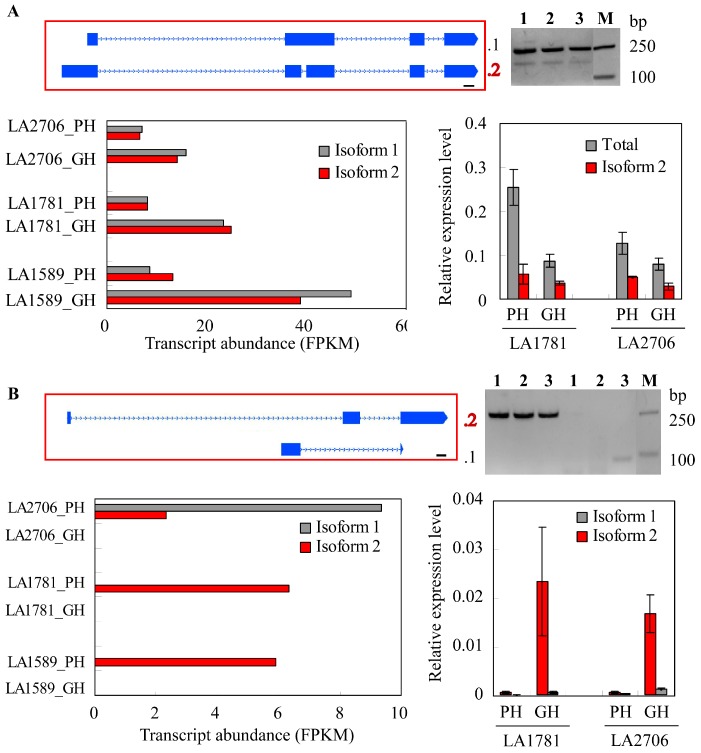

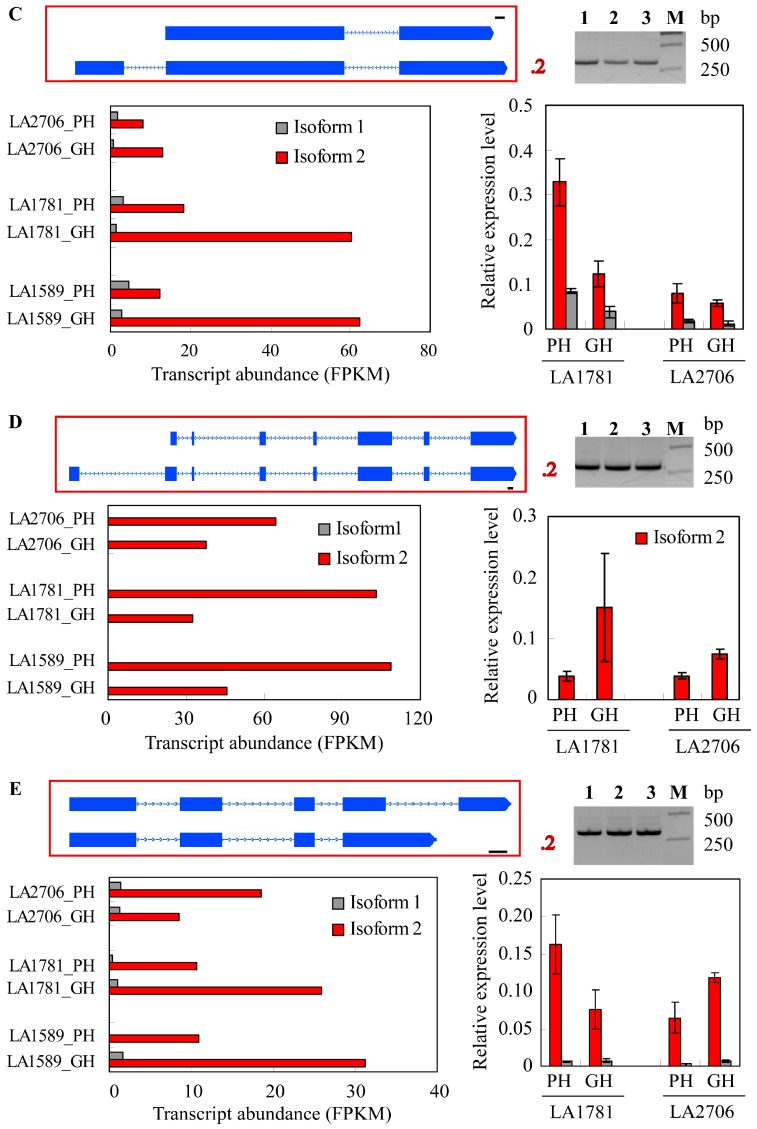
Validation of five isoforms showing differential expression in response to distinct growth conditions. The differentially expressed transcript isoforms of genes encoding a CCT (CONSTANS, CO-like, and TOC1) motif family protein (*Solyc03g083400*, (**A**)); a R2R3 MYB transcription factor (*Solyc05g008240*, (**B**)); the ethylene receptor ETR4 (*Solyc06g053710*, (**C**)); Protein-P-II uridylyltransferase (*Solyc06g059800*, (**D**)) and Aluminum-induced protein-like (*Solyc02g078500*, (**E**)) were verified by RT-PCR and qRT-PCR using total RNA samples collected from GH and PH grown seedlings at 5 days post germination. In (**A**–**E**), the upper panel is showing the intron–exon structures of the two isoforms (left) and agarose gel images of RT-PCR analysis using isoform-specific primers (right). The lower panel is showing the transcript abundance revealed by RNA-seq (left) and qRT-PCR (right). The splice variants were labeled as .2 (isoform 2), whereas the annotated transcripts (isoform 1) were indicated by .1 (in **A**,**B**) or not particularly labeled (in **C**–**E**) at the right side of the gene structures, which introns and exons are represented by dotted lines and blue boxes. The numbers 1, 2 and 3 above each gel image in (**A**–**E**) represent cDNA samples from LA1589, LA1781 and LA2706 seedlings. M, the DNA marker with the band sizes, is indicated at the right side.

We then validated the AS events and the expression level of the splice variants for the abovementioned five genes by reverse transcriptase PCR (RT-PCR) using GH and PH seedlings. The putative photosystem I reaction center subunit *Solyc01g007350* was not included because it was likely misannotated—a very long transcript (6722 bp) was found that spanned several annotated genes including *Solyc01g007350*. As shown in Figure 5, the AS events were confirmed by isoform-specific RT-PCR. In addition, further quantification of variant abundance in the seedlings also confirmed the expression levels estimated by RNA-seq.

### 2.4. Response of Alternative Splicing to Environments

Previous studies indicate that AS is regulated by light and stresses [11,25,26,27]. The growth conditions under PH and GH vary considerably although both are optimal to plant growth. We identified 238 genes showing differential splicing in response to growth condition changes, of which 44, 71 and 129 were from LA1589, LA1781 and LA2706 (Table S8). When further classifying the 238 genes based on their putative functions involved, the number of genes involved in growth and development, photosynthesis and response to light, and RNA processing and splicing had the largest differences among the three genetic backgrounds (Table 2). Among these differential splicing genes, seven genes are likely involved in chlorophyll biosynthesis and chloroplast development, photosynthesis light-harvesting and electron transfer. For example, the coproporphyrinogen III oxidase Solyc10g005110 shares high similarity with *Arabidopsis* LESION INITIATION 2 (LIN2), a key enzyme in chlorophyll biosynthesis in [47], and Solyc12g037930 is the Arabidopsis homolog VARIEGATED 3 (VAR3) that has been shown to be required for chloroplast development [48]. Two differential splicing genes—*Solyc05g013750* and *Solyc12g005340*—are likely involved in plant adaptation to light quality because they share high similarity with the respective *Arabidopsis* homologs *Mitogen-activated protein kinase phosphatase 1* (*MKP1*) and *phytochrome A signal transduction 1* (*PAT1*), which play roles in plant response to UV-B and far-red light mediated by *phytochrome A* (*PHY A*) [49,50,51,52]. Previous studies have shown that many splicing factors were regulated by AS [28,30,39,53,54,55,56,57]; we also found that there are three splicing factors including serine/arginine rich protein SR30 (Solyc01g099810), LHP1-interacting factor 2 (LIF2, Solyc02g088720) and a C1D family protein (Solyc07g008750), whose pre-mRNA were regulated by AS. Interestingly, we detected three differential splicing genes involved in the brassinosteroids (BR) signaling pathway; they all encode protein kinases similar to Arabidopsis BIR1 (Solyc07g006480), HERK1 (Solyc05g013300) and BSK2 (Solyc01g080880). This implies that BR signaling is regulated by AS in response to environmental changes.

**Figure 6:**
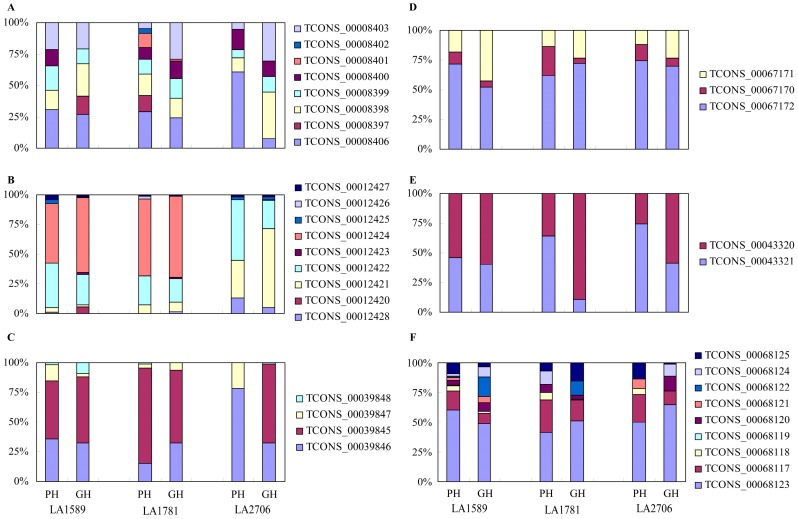
Isoform expression patterns of six representative differential splicing genes. (**A**) Isoform expression pattern of the differential splicing gene *SR30* (*Solyc01g099810*). The transcript abundance for six of the nine *SR30* isoforms showed variations in response to growth condition changes and most of them were more abundant in PH seedlings; (**B**) Isoform expression pattern of the differential splicing gene *Solyc02g088720*, similar to *Arabidopsis LIF2.* Only two of its nine mRNA isoforms were predominantly expressed under a particular growth condition; (**C**) Isoform expression pattern of the differential splicing gene *Solyc07g008750*, encoding a protein similar to Exosome-associated factor Rrp47/C1D family member. Under particular conditions, two isoforms contributed to the majority of transcript abundance; (**D**) Isoform expression pattern of the differential splicing gene *PAT1* (*Solyc12g005340*); (**E**) Isoform expression pattern of the differential splicing gene *Lhcb2.1* (*Solyc07g047850*); (**F**) Isoform expression pattern of the differential splicing gene *Solyc12g021280*, similar to Arabidopsis *STT7 HOMOLOG STN7* (*STN7*).

To gain some insight into the AS response to environmental changes, we checked the expression of the splice variants of three splicing factors and three photosynthesis-related genes that were identified as differential splicing genes. These genes generated one to eight splice variants and most of them had no significant changes in expression levels between the two growth conditions, except one splice variant of the *LIF2*-like gene *Solyc02g088720*, which was more abundant in the Moneymaker seedlings grown under GH condition (Figure 6). At gene level, only the C1D gene family member *Solyc07g008750* showed differential expression between the two growth conditions. Furthermore, it is also noticeable that for each gene, two to three splice variants were expressed at relatively high levels. Thus, differential splicing of these splicing factors and photosynthetic genes was regulated on few main AS events. On further analysis of the major splice variants of the six genes, we found that most of them contain PTC and few encode novel proteins.

## 3. Discussion

Transcriptome dynamics help plants to adapt to their changing environments. In this study, we compared the gene expression and alternative splicing of young tomato seedlings grown in two distinct growth systems—phytotron and plastic greenhouse. The two environmental conditions represent two typical plant growth systems; phytotron provides well-controlled environmental factors of light, temperature and humidity, whereas the plastic greenhouse, a common vegetable growth system world-wide, has a complex environment with fluctuating solar radiation and temperature [58]. Our data revealed transcriptome features under different growth conditions and we also identified candidate genes responsible for plant adaptation to changing environments in tomato. The results not only provide useful information to predict gene expression in tomato plants grown in a plastic greenhouse but also help understand the transcriptional and posttranscriptional regulation of plant developmental plasticity.

### 3.1. Genotype-Dependent AS Regulation by Distinct Environments

Our data indicate that the frequency and the pattern of alternative splicing are genotype-dependent and are affected by growth environments, which is in agreement with previous results that AS is dependent on cell types and developmental stages [11,15,27,28,53]. Although the numbers of the expressed genes were similar among the three genotypes (two *S. pimpinellifolium* accessions and one cultivated tomato) and between the two growth conditions, the numbers of splice variants and multi-exon genes that underwent AS varied considerably; more splice variants and higher AS frequencies were detected in the two wild species accessions—LA1589 and LA1781—than in the cultivar Moneymaker. Furthermore, there were almost no common genes among the three genotypes that showed differential splicing in response to distinct growth conditions, also confirming the genotypic specificity of AS responses to changing environments. Few splice variants were identified in the cultivar Moneymaker; this is likely due to the sequence divergence between cultivated tomato and *S. pimpinellifolium*. We compared our RNA-seq reads to the predicted transcripts from the reference genome of the cultivated tomato Heinz1706, which has 0.6% nucleotide difference with *S. pimpinellifolium* LA1589 [59], which may slightly affect the AS prediction accuracy.

Higher frequencies of IR-type AS events detected in GH seedlings indicate that the fluctuating environmental conditions in GH enhance intron retention. Thus, the direction of AS changes may be significantly influenced by growth conditions. Because the main environmental differences between GH and PH are most likely the fluctuations in light quality and temperature, we reason that light and/or temperature triggered the directional changes in alternative splicing. Indeed, there were twelve genes involved in photosynthesis and light signaling, showing distinct splicing patterns between the two growth conditions. Upon exposure to light, increase of the IR-type AS events has also been observed in *P.patens* and Arabidopsis [30,33]. Since IR events often introduce premature termination codons (PTC) into the spliced mRNA transcript [11,17,60,61], many of these splice variants are likely degraded rapidly through a nonsense-mediated mRNA decay (NMD) mechanism [62,63,64,65,66]. Thus, the coupled AS-NMD may play an important role in regulation of gene expression in response to fluctuating environments.

Consistent with the abovementioned species- or genotype-specific pattern of AS regulation, we found that the expression of splice variants was also genotype specific. Although there were 665 splice variants having significantly different expression levels between the two growth conditions, only five of them were modulated in all the seedlings from LA1589, LA1781 and Moneymaker. Even within the same species, few splice variants in LA1589 and LA1781 showed consistently differential expression in response to changing growth conditions, indicating that transcriptional response of splice variants to environmental changes is specific to genetic background or genotype.

### 3.2. Transcriptome Dynamics in Response to Environmental Fluctuations

Many crop traits are significantly affected by environments, showing plasticity in response to different growth conditions. The phenotypic plasticity has been associated with transcriptome dynamics under fluctuating environmental conditions [3,7,67]. In this study, we found 5%–21% of the expressed genes in the cultivar Moneymaker and the two accessions of its closest wild relatives were responsive to the changing growth conditions. Previous studies have identified similar and even higher percentages of genes showing differential expression in response to fluctuating environments under field conditions [3,5,7,9]. For example, Dal Santo et al. reported that 5% of protein-coding genes expressed in grapevine during berry development were modulated by variations in growth conditions [7]. A comprehensive transcriptome analysis of rice (*Oryza sativa*) under field conditions revealed that more than 40% of all the expressed rice genes responded to fluctuating temperature, solar radiation and circadian rhythms [8]. We may underestimate the transcriptome plasticity because, to make experiments more practicable and to reduce the variations between replicates, we used the same kind of soil to grow these seedlings and did not test the impacts of nutrient and water supply. Nevertheless, these results indicate that a considerable portion of plant transcriptome is modulated by environmental fluctuations in nature.

Further identification of common genes responding to fluctuating field environments will be useful for predicting plant gene expression in the field and also help the transfer of the laboratory-based knowledge to the real agriculture problems. In this study, we found about one hundred genes responding to the changing growth conditions consistently in the cultivated tomato Moneymaker and its two closest wild relatives. Some of these environmentally-regulated genes involve photosynthesis and development, indicating that solar radiation and/or temperature have profound impacts on gene expression in response to environmental fluctuations because they have the most noticeable difference between the two growth conditions. It has been shown in rice that field type and macroclimate impose significant effects on gene expression involved in photosynthesis and development under field conditions [8]. Therefore, solar radiation (and likely temperature also) is one of the most influential environmental factors triggering transcriptional response. The notion is further supported by the fact that there are twelve photosynthetic and light signaling genes showing differential splicing in response to changing growth environments.

Finally, because a plastic greenhouse is being used for vegetable production globally, our results may also provide transferable knowledge to understand the interaction between the genetic composition and the environmental perturbations that is crucial for modern crop production under the trend of global climate changes.

## 4. Materials and Methods

### 4.1. Plant Growth Conditions

The closest wild ancestor to cultivated tomato *S. pimpinellifolium* LA1589 and LA1781 and the cultivar Moneymaker (LA2706) were kindly provided by the Tomato Genetics Resource Center at University of California, USA. Seeds were sowed directly in blonde peat (Pindstrup Mosebrug A/S, Ryomgaard, Denmark) and germinated in phytotrons and a plastic greenhouse. The phytotron used in this study was maintained at 20–25 °C with a humidity of 70%–80% and the seedlings/plants were illuminated daily by 150 mE·m^−2^·s^−1^ light for 16 h. By comparison, seeds were also germinated in a plastic greenhouse located in Shanghai, which is a standard solar greenhouse, like those used for vegetable production in China. The plastic greenhouse was under solar radiation, and during the experiments (April 2016), the local climate had an average daily temperature of 21.3 °C (lowest at 9.8 °C and highest at 35.8 °C) and the relative humidity of 64.4% (33.7%–83.2%).

### 4.2. RNA Sequencing, Read Mapping and Transcript Assembly

Total RNA was isolated from 5-days-old whole seedlings using Trizol reagent (Thermo Fisher Scientific, Carlsbad, CA, USA) based on the methods described previously [42]. RNA-seq libraries were generated from 1 μg total RNA using NEBNext Ultra Directional RNA Library Prep Kit for Illumina (E7420L, New England BioLabs, Ipswich, MA, USA) and were sequenced on an Illumina Hiseq2500 system using Hiseq SBS Kit V3 (Illumina Inc., San Diego, CA, USA).

The quality of raw reads was first evaluated by FastQC (available online: www.bioinformatics.babraham.ac.uk/projects/fastqc). After adapter sequences were trimmed using FASTX Toolkit (version 0.0.13; available online: hannonlab.cshl.edu/fastx_toolkit/index.html), low quality reads with a quality score below 20 were removed by Trimmomatic (version 0.36) [68]. Because the cultivated tomato and its closest wild relatives *S. pimpinellifolium* have only 0.6% nucleotide divergence in genome sequences [59], we received clean reads from LA1589 and LA1781 as well as those from the cultivar cv. Moneymaker were mapped to the reference genome of the cultivar Heinz1706 (version ITAG2.5 from Sol Genomics Network (SGN); available online: https://solgenomics.net/) by Tophat program v.2.1.1 [69]. The main parameters used are as followed: -N 3-read-edit-dist 3 –I 35 –I 120000—library-type fr-firststrand. Transcripts were assembled from the mapped reads using the Cufflinks program (version 2.2.1) guided by the reference annotation [14]. Then, the Cuffdiff tool in the program was used to select genes or isoforms showing differential expression (adjusted *p*-value of 0.05 or less) in response to different growth conditions. We also used the Cuffdiff program to test the significance of the alternative splicing changes between GH and PH growth conditions.

### 4.3. Identification of AS Events

We extracted AS events from the GTF files generated by Cufflinks using the online tool ASTALAVISTA program (available online: http://genome.crg.es/astalavista/) [70].

### 4.4. Gene Ontology Enrichment Analysis of Differentially Expressed Genes and Isoforms

Enrichment analysis of gene ontology terms was conducted on differentially expressed genes or isoforms using the online tool AgriGO (available online: http://bioinfo.cau.edu.cn/agriGO/analysis.php) [45]. A statistical hypergeometric test and multi-test correction by the Bonferroni method were applied in the singular enrichment analysis of gene ontology terms. Functional categories of GO terms with a false discovery rate (FDR) smaller than 0.05 (adjusted *p*-value < 0.05) were considered as significantly enriched.

### 4.5. Validation of Isoform Expression by RT-PCR and Quantitative RT-PCR (qRT-PCR)

Total RNA was extracted from whole seedlings at five days post germination using the Trizol reagent (Invitrogen, Carlsbad, CA, USA) following the manufacturer’s instructions. RNA samples were then treated with RNase-free DNase (New England BioLabs, Ipswich, MA, USA) for 10 min. An amount of 1 μg of the DNase-treated total RNA per sample was used to synthesize first strand cDNA using a ProtoScript^®^ II First Strand cDNA Synthesis Kit (New England BioLabs, Ipswich, MA, USA) following the manufacturer’s protocols. Twenty percent of the reverse transcription products were subjected to PCR analysis.

Real-time RT-PCR analysis was performed on three independent biological replicates using AceQ^®^ qPCR SYBR Green Master Mix (Vazyme Biotech Co., Ltd., Nanjing, China), and on an ABI Applied Biosystems StepOnePlus machine according to the manufacturer’s instructions. The transcript level of the tomato *SleIF4α6* gene was used as a reference to calculate the relative expression values of each target gene. Primer information can be found in Table S9.

## 5. Conclusions

Through RNA-seq analysis of young seedlings grown under two distinct environmental conditions, we revealed profound impacts of growth conditions on transcriptome dynamics and complexity. Environments not only impact expression of a considerable number of genes, but also affect alternative splicing of many multiexon genes in a genotype-specific manner. Our results provide useful information to further our understanding on gene regulation of environmentally sensitive biological processes in plants.

## Figures and Tables

**Table 1:** Environmentally-sensitive genes involved in photosynthesis and light signaling.

ITAG2.4 ID	Fold Changes (log2)			Description
	LA1589	LA1781	LA2706	
Solyc11g068430	−2.95	−2.74	−1.03	Ferredoxin
Solyc06g069730	−1.30	−3.34	−1.28	Chlorophyll a–b binding protein 4, chloroplastic
Solyc05g056050	−1.22	−2.34	−1.43	Chlorophyll a–b binding protein
Solyc08g006930	−1.16	−1.52	−1.09	Photosystem I reaction center subunit X psaK
Solyc12g006140	−1.14	−3.16	−1.48	Chlorophyll a–b binding protein 37
Solyc02g077240	−5.43	−3.96	−2.01	Pyruvate decarboxylase
Solyc10g076510	−3.88	−2.02	−1.04	Pyruvate decarboxylase
Solyc06g048410	−1.76	−2.15	−1.05	Superoxide dismutase
Solyc03g120990	−1.24	−2.50	−2.12	Malic enzyme; Malic enzyme, NAD-binding
Solyc01g095140	−2.80	−2.34	−1.04	Late embryogenesis abundant protein; response to light
Solyc10g047530	−1.29	−2.80	−1.28	Phototropic-responsive NPH3 family protein
Solyc01g109700	1.00	1.31	1.01	Transcription factor; Helix-loop-helix DNA-binding
Solyc06g007180	1.78	4.10	3.72	Asparagine synthase (Glutamine-hydrolyzing)
Solyc05g008240	infinite *	infinite	infinite	myb3 like; similar to R2R3-myb anthocyanin repressor

* Infinite indicates that expression was only detected in GH seedlings. Fold change was expressed in log2(GH/PH), the positive and negative values of which indicate higher or lower expression in the GH seedlings.

**Table 2:** Functional categories of genes showing differential splicing in response to changing growth environments.

Functional Category	*S. Pimpinellifolium*		*S. Lycopersicum*
	LA1589	LA1781	LA2706
DNA modification	2 (4.5) *	2 (2.8)	3 (2.3)
RNA metabolism	3 (6.8)	8 (11.3)	14 (10.9)
Protein biosynthesis and modification	10 (22.7)	11 (15.5)	14 (10.9)
Response to stresses	6 (13.6)	7 (9.9)	8 (6.2)
Growth and development	5 (11.4)	5 (7.0)	18 (14.0)
Photosynthesis and light signaling	-	4 (5.6)	5 (3.9)
miscellaneous	3 (6.8)	8 (11.3)	14 (10.9)
Unknown	44 (34.1)	26 (36.6)	53 (41.1)
Total	44 (100)	71 (100)	129 (100)

* The numbers represent gene numbers or percentages (in parentheses) in particular functional categories.

## Supplementary Materials

Supplementary materials can be found at www.mdpi.com/1422-0067/18/3/475/s1.
